# Activation of Bt Protoxin Cry1Ac in Resistant and Susceptible Cotton Bollworm

**DOI:** 10.1371/journal.pone.0156560

**Published:** 2016-06-03

**Authors:** Jizhen Wei, Gemei Liang, Bingjie Wang, Feng Zhong, Lin Chen, Myint Myint Khaing, Jie Zhang, Yuyuan Guo, Kongming Wu, Bruce E. Tabashnik

**Affiliations:** 1 State Key Laboratory for Biology of Plant Diseases and Insect Pests, Institute of Plant Protection, Chinese Academy of Agricultural Sciences, Beijing 100193, China; 2 Department of Entomology and BIO5 Institute, University of Arizona, Tucson, Arizona 85721, United States of America; Institute of Vegetables and Flowers, Chinese Academy of Agricultural Sciences, CHINA

## Abstract

Crystalline (Cry) proteins from *Bacillus thuringiensis* (Bt) are used extensively for insect control in sprays and transgenic plants, but their efficacy is reduced by evolution of resistance in pests. Here we evaluated reduced activation of Cry1Ac protoxin as a potential mechanism of resistance in the invasive pest *Helicoverpa armigera*. Based on the concentration killing 50% of larvae (LC_50_) for a laboratory-selected resistant strain (LF120) divided by the LC_50_ for its susceptible parent strain (LF), the resistance ratio was 1600 for Cry1Ac protoxin and 1200 for trypsin-activated Cry1Ac toxin. The high level of resistance to activated toxin as well as to protoxin indicates reduced activation of protoxin is not a major mechanism of resistance to Cry1Ac in LF120. For both insect strains, treatment with either the trypsin inhibitor N-a-tosyl-L-lysine chloromethyl ketone (TLCK) or the chymotrypsin inhibitor N-a-tosyl-L-phenylalanine chloromethyl ketone (TPCK) did not significantly affect the LC_50_ of Cry1Ac protoxin. Enzyme activity was higher for LF than LF120 for trypsin-like proteases, but did not differ between strains for chymotrypsin-like proteases. The results here are consistent with previous reports indicating that reduced activation of protoxin is generally not a major mechanism of resistance to Bt proteins.

## Introduction

Insecticidal proteins from the bacterium *Bacillus thuringiensis* (Bt) have been used extensively for pest control in sprays and genetically engineered crops [1–3]. These Bt proteins kill some major insect pests, but are not toxic to most other organisms, including humans [4–7]. The area planted worldwide to transgenic crops producing Bt proteins increased to 84 million hectares (ha) in 2015, with a cumulative total of >732 million ha since 1996 [1]. Although Bt crops have provided substantial environmental and economic benefits [8–14], evolution of pest resistance to Bt proteins can reduce or eliminate these benefits [15–18].

Understanding the mode of action and the mechanisms of resistance to Bt proteins can help to enhance and sustain their efficacy against pests. Many studies have focused on the closely related crystalline Bt proteins Cry1Ab and Cry1Ac, which kill lepidopteran pests and are produced by widely adopted transgenic Bt corn, cotton, and soybeans [1–3, 19–21]. Models of Bt mode of action agree that the full-length forms of Cry1Ab and Cry1Ac proteins called protoxins are converted by insect midgut proteases to activated toxins that bind to insect midgut receptors, eventually leading to death of susceptible insects [19–21]. This activation entails removal of approximately 40 amino acids from the amino terminus and 500 amino acids from the carboxyl terminus, converting the protoxins of approximately 130 kDa to activated toxins of approximately 55 to 65 kDa [19–21].

Whereas the established “classical” model for Cry1A mode of action asserts that only the activated toxins of ca. 55 to 65 kDa can bind to receptors and kill insects [19–21], some recent evidence supports a “dual” model in which activated toxins exert toxicity via a primary pathway and intact protoxins or some other portion of protoxins exert toxicity via a different pathway [22–24]. Consistent with both models, reduced conversion of protoxin to activated toxin by midgut proteases can cause greater resistance to protoxins than activated toxins [24–33].

Here we examined activation of Cry1Ac protoxin by proteases in resistant and susceptible strains of *Helicoverpa armigera*, one of the world’s most damaging crop pests [34]. After nearly two decades of exposure to transgenic cotton plants producing Cry1Ac, field populations of *H*. *armigera* have remained susceptible to Cry1Ac in Australia [35] and have shown small, but significant increases in resistance to Cry1Ac in China [36–38]. In the laboratory, many strains of *H*. *armigera* have been selected for high levels of resistance to Cry1Ac [29, 37, 39–41].

We analyzed two previously described strains from China: the susceptible LF strain and the resistant LF120 strain [29, 40–42]. The LF120 strain had >1000-fold lab-selected resistance to Cry1Ac and was derived from the LF strain via a series of progressively more resistant strains [29, 40–42]. We evaluated activation of Cry1Ac protoxin by two types of midgut serine proteases: trypsin-like proteases, which are inhibited by N-a-tosyl-L-lysine chloromethyl ketone (TLCK), and chymotrypsin-like proteases, which are inhibited by N-a-tosyl-L-phenylalanine chloromethyl ketone (TPCK). The results imply that trypsin-like proteases were more important than chymotrypsin-like proteases in activation of Cry1Ac protoxin and that reduced activation of Cry1Ac protoxin has at most a minor role in resistance to Cry1Ac of the LF120 strain of *H*. *armigera*.

## Results

### Effects of Cry1Ac protoxin and activated toxin on mortality of resistant (LF120) and susceptible (LF) larvae

Relative to its susceptible parent strain (LF), the LF120 strain of *H*. *armigera* was highly resistant to Cry1Ac protoxin and activated toxin (Table 1). The resistance ratio, calculated as the concentration of Cry1Ac killing 50% (LC_50_) for LF120 larvae divided by the LC_50_ for LF larvae, was 1600 for protoxin and 1200 for activated toxin (Table 1). For each strain, based on the conservative criterion of non-overlap between the 95% fiducial limits (FL), LC_50_ values did not differ significantly between Cry1Ac protoxin and activated toxin (Table 1).

**Table 1:** Effects of Cry1Ac protoxin and activated toxin on mortality of resistant (LF120) and susceptible (LF) larvae of *H*. *armigera*.

Strain	Treatment	Slope ± SE^a^	LC_50_ (95% FL)^b^	RR^c^
LF	Protoxin	0.95 ± 0.11	13.0 (6.9–21)	1.0
LF120	Protoxin	0.58 ± 0.08	20,800 (12,000–47,000)	1600*
LF	Activated toxin	1.18 ± 0.13	12.0 (7.5–17)	1.0
LF120	Activated toxin	0.56 ± 0.09	14,800 (7400–32,000)	1200*

^a^ Slope of the concentration-mortality line and its standard error

^b^ Concentration killing 50% with 95% fiducial limits in ng Cry1Ac per cm^2^ diet

^c^ Resistance ratio, LC_50_ for LF120 divided by LC_50_ for LF.

*Asterisks indicate significantly higher LC_50_ for LF120 than LF by the conservative criterion of non-overlap of 95% FL.

### Effects of protease inhibitors on toxicity of Cry1Ac protoxin to resistant and susceptible larvae

The trypsin inhibitor TLCK and the chymotrypsin inhibitor TPCK did not significantly affect the LC_50_ of Cry1Ac protoxin for LF or LF120 (Table 2). In the protease inhibitor experiment (Table 2), all larvae were pre-treated with the solvent DMSO (see Methods). A comparison of the results between Table 1 (no DMSO) and Table 2 (DMSO) shows that DMSO did not significantly affect the LC_50_ values of Cry1Ac protoxin for either strain. In the protease inhibitor experiment, resistance ratios for LF120 relative to LF were 1200 for Cry1Ac protoxin, 690 for Cry1Ac protoxin + TLCK, and 890 for Cry1Ac protoxin + TPCK (Table 2).

**Table 2:** Effects of protease inhibitors TLCK and TPCK on toxicity of Cry1Ac protoxin to resistant (LF120) and susceptible (LF) larvae of *H*. *armigera*.

Strain	Treatment^a^	Slope ± SE^b^	LC_50_ (95% FL)^c^	RR^d^	IR^e^
LF	Protoxin	2.32 ± 0.26	11.6 (2.6–16)	1.0	NA
LF	Protoxin + TLCK	1.61 ± 0.26	27.2 (11–49)	1.0	2.3
LF	Protoxin + TPCK	1.90 ± 0.26	20.1 (14–27)	1.0	1.7
LF120	Protoxin	0.70 ± 0.09	13,600 (9000–22,000)	1200*	NA
LF120	Protoxin + TLCK	0.74 ± 0.09	13,900 (6400–43,000)	690*	1.0
LF120	Protoxin + TPCK	0.63 ± 0.09	24,200 (15,000–48,000)	890*	1.8

^a^ All larvae were pre-treated with the solvent DMSO (see Methods).

^b^ Slope of the concentration-mortality line and its standard error

^c^ Concentration killing 50% with 95% fiducial limits in ng Cry1Ac per cm^2^ diet

^d^ Resistance ratio, LC_50_ for LF120 divided by LC_50_ for LF.

*Asterisks indicate significantly higher LC_50_ for LF120 than LF by the conservative criterion of non-overlap of 95% FL.

^e^ Inhibition ratio (IR), LC_50_ of Cry1Ac protoxin with inhibitor divided by the LC_50_ of Cry1Ac protoxin without inhibitor.

### Effects of protease inhibitors on activation of Cry1Ac by trypsin, chymotrypsin, and midgut extract from susceptible larvae

**Fig 1:**
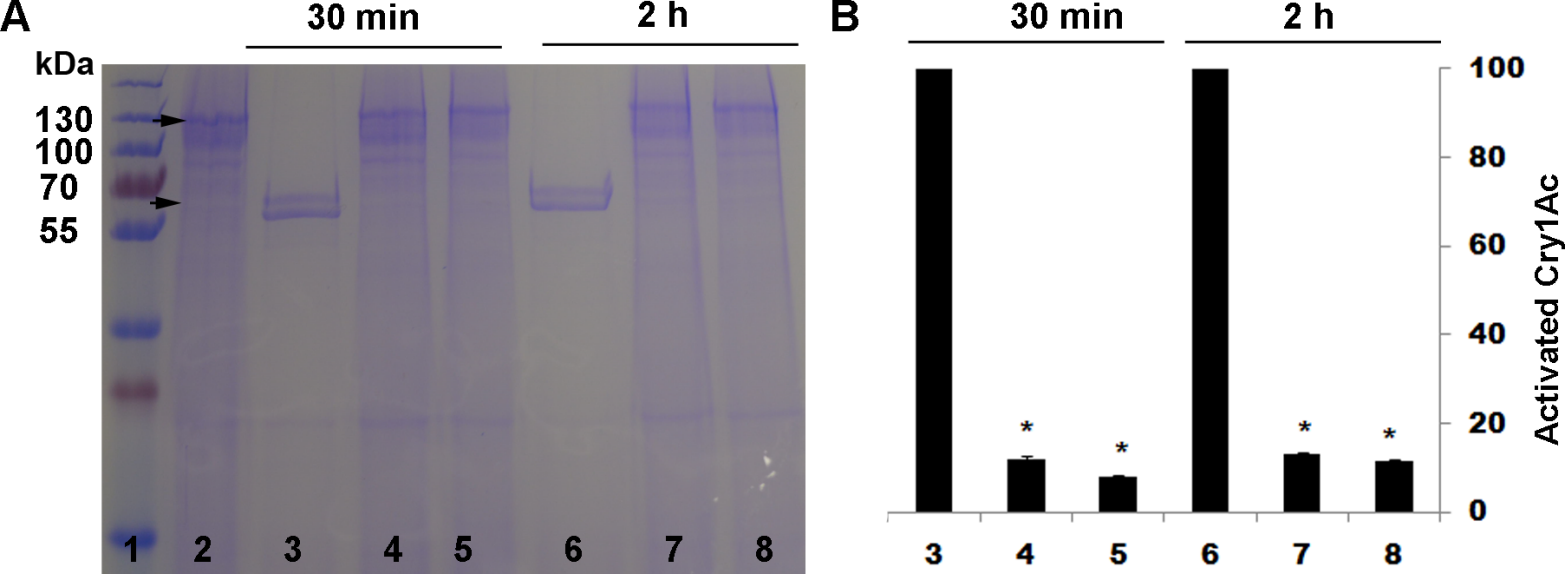
Activation of Cry1Ac protoxin by trypsin with and without the trypsin inhibitor TLCK. (A) Representative SDS-PAGE gel. (B) Percentage activation (mean and SE) based on optical density of the activated toxin band (65 kDa) relative to the band with only protoxin (lane 2) calculated by Image J quantification from three replicates. Asterisks indicate significantly lower activation with inhibitor than without for a given incubation period: 30 min for lanes 4 and 5 versus lane 3 and 2 h for lanes 7 and 8 versus lane 6 (t-tests, P < 0.05). Lane 1, molecular weight markers (kDa); Lane 2, Cry1Ac protoxin; Lanes 3 and 6, Cry1Ac protoxin and trypsin; Lanes 4 and 7, Cry1Ac protoxin and 10:1 trypsin + TLCK; Lanes 5 and 8, Cry1Ac protoxin and 1:1 trypsin + TLCK.

**Fig 2:**
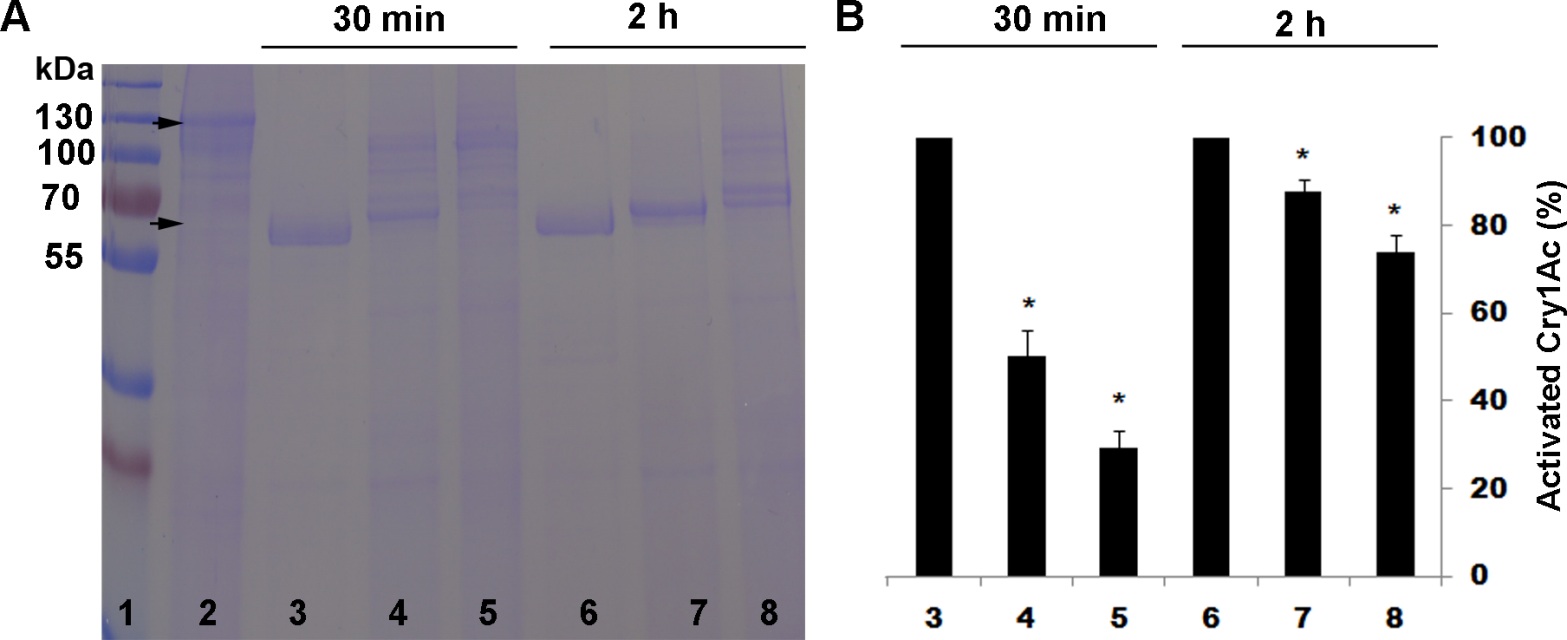
Activation of Cry1Ac protoxin by chymotrypsin with and without the chymotrypsin inhibitor TPCK. (A) Representative SDS-PAGE gel. (B) Percentage activation (mean and SE) based on optical density of the activated toxin band (65 kDa) relative to the band with only protoxin (lane 2) calculated by Image J quantification from three replicates. Asterisks indicate significantly lower activation with inhibitor than without for a given incubation period: 30 min for lanes 4 and 5 versus lane 3 and 2 h for lanes 7 and 8 versus lane 6. Lane 1, molecular weight markers (kDa); Lane 2, Cry1Ac protoxin; Lanes 3 and 6, Cry1Ac protoxin and chymotrypsin; Lanes 4 and 7, Cry1Ac protoxin and 10:1 chymotrypsin + TPCK; Lanes 5 and 8, Cry1Ac protoxin and 1:1 chymotrypsin + TPCK.

**Fig 3:**
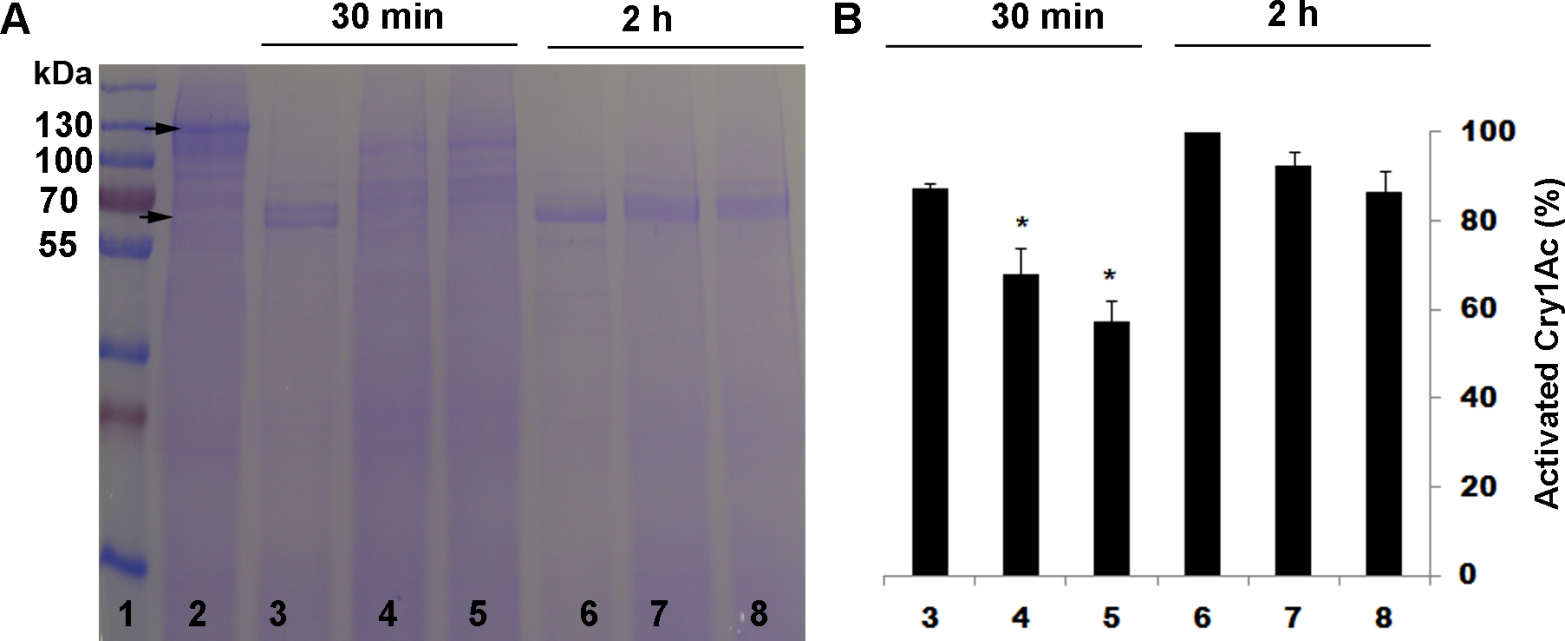
Activation of Cry1Ac protoxin by midgut extract from the susceptible strain LF of *H*. *armigera* with and without the trypsin inhibitor TLCK. (A) Representative SDS-PAGE gel. (B) Percentage activation (mean and SE) based on optical density of the activated toxin band (65 kDa) relative to the band with only protoxin (lane 2) calculated by Image J quantification from three replicates. Asterisks indicate significantly lower activation with inhibitor than without for a given incubation period: 30 min for lanes 4 and 5 versus lane 3 and 2 h for lanes 7 and 8 versus lane 6 (t-tests, P < 0.05). Lane 1, molecular weight markers (kDa); Lane 2, Cry1Ac protoxin; Lanes 3 and 6, Cry1Ac protoxin and midgut extract; Lanes 4 and 7, Cry1Ac protoxin and 10:1 midgut extract + TLCK; Lanes 5 and 8, Cry1Ac protoxin and 1:1 midgut extract + TLCK.

**Fig 4:**
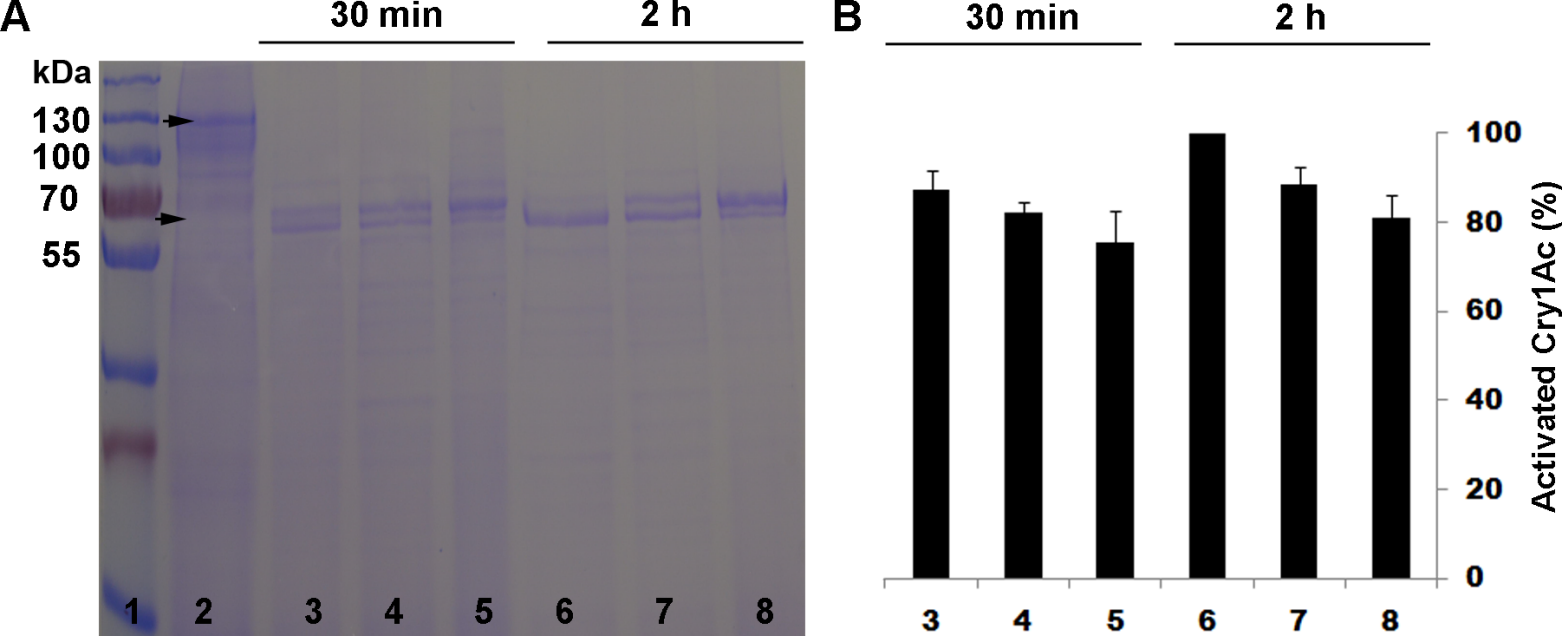
Activation of Cry1Ac protoxin by midgut extract from the susceptible strain LF of *H*. *armigera* with and without the chymotrypsin inhibitor TPCK. (A) Representative SDS-PAGE gel. (B) Percentage activation (mean and SE) based on optical density of the activated toxin band (65 kDa) relative to the band with only protoxin (lane 2) calculated by Image J quantification from three replicates. For each incubation period (30 min for lanes 3–5 and 2 h for lanes 6–8), the inhibitor TPCK had no significant effect on activation (t-tests, P > 0.05). Lane 1, molecular weight markers (kDa); Lane 2, Cry1Ac protoxin; Lanes 3 and 6, Cry1Ac protoxin and midgut extract; Lanes 4 and 7, Cry1Ac protoxin and 10:1 midgut extract + TPCK; Lanes 5 and 8, Cry1Ac protoxin and 1:1 midgut extract + TPCK.

As expected, after both 30 min and 2 h of incubation, the trypsin inhibitor TLCK significantly reduced activation of Cry1Ac protoxin by trypsin (Fig 1) and the chymotrypsin inhibitor TPCK significantly reduced activation of Cry1Ac protoxin by chymotrypsin (Fig 2). By contrast, in experiments where Cry1Ac protoxin was activated by a midgut extract from susceptible LF larvae, TLCK significantly reduced activation after 30 min of incubation but not after 2 h of incubation (Fig 3), and TPCK did not significantly reduce activation after either 30 min or 2 h of incubation (Fig 4).

### Protease activity in susceptible and resistant larvae

**Fig 5:**
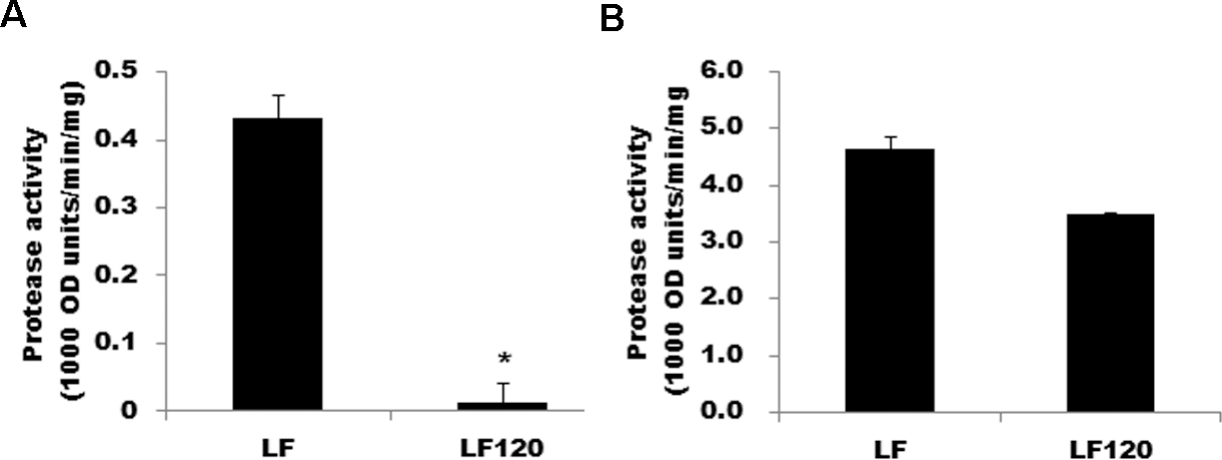
Protease activity in fifth instars of the susceptible LF strain and resistant LF120 strain of *H*. *armigera*. (A) Trypsin-like proteases. (B) Chymotrypsin-like proteases. The asterisk indicates significantly lower trypsin-like activity for LF120 than LF. Chymotrypsin activity did not differ significantly between strains.

The activity of trypsin-like proteases was 33-fold higher in the susceptible strain than in the resistant strain (P = 0.02), but the activity of chymotrypsin-like proteases did not differ significantly between strains (P = 0.55) (Fig 5).

### Activation of Cry1Ac protoxin by midgut extracts from resistant and susceptible larvae

**Fig 6:**
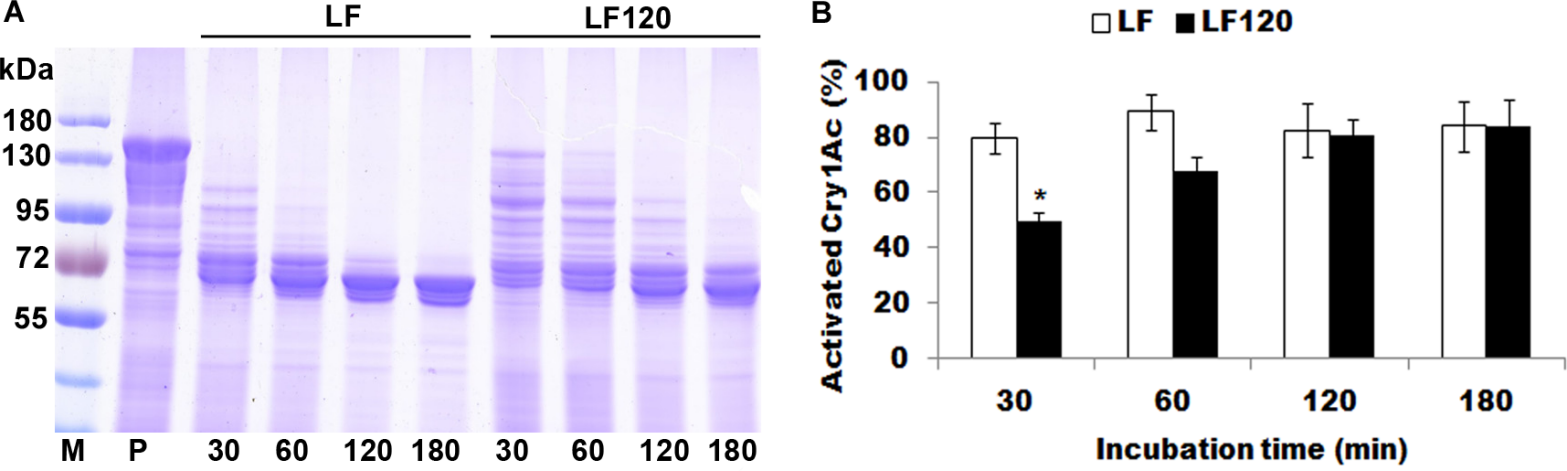
Time course of the activation of Cry1Ac protoxin by midgut extracts from the susceptible LF strain and resistant LF120 strain of *H*. *armigera*. (A) Representative SDS-PAGE gel. (B) Percentage activation (mean and SE) based on optical density of the activated toxin band (65 kDa) relative to the band with only protoxin (P) calculated by Image J quantification from three replicates. M: Protein marker; P: Only protoxin; 30, 60, 120 and 180 were the incubation time points (min). The asterisk indicates significantly lower activation for LF120 than LF at 30 min. Activation did not differ significantly between strains at 60, 120, and 180 min.

The percentage of Cry1Ac protoxin activated by midgut extracts was significantly higher for the susceptible strain than the resistant strain at 30 min (P = 0.014, Fig 6), but did not differ significantly between strains at 60, 120, and 180 min (P = 0.063, 0.72 and 0.98 at 60, 120 and 180 min, respectively, Fig 6).

### Induction of proteases by Cry1Ac in susceptible larvae

In fifth instar of the susceptible LF strain of *H*. *armigera*, feeding for 12 h on diet treated with Cry1Ac protoxin increased the activity of both trypsin-like and chymotrypsin-like proteases (S1 Fig). The activity of each type of protease increased linearly as the concentration of Cry1Ac increased (S1 Fig). Relative to untreated diet, the highest concentration of protoxin tested (13 μg Cry1Ac per g diet) caused a 7.9-fold increase in the activity of trypsin-like proteases and a 3.6-fold increase in the activity of chymotrypsin-like proteases (S1 Fig).

## Discussion

The results here show that relative to the susceptible LF strain of *H*. *armigera*, the LF120 strain had a resistance ratio of 1600 for Cry1Ac protoxin and 1200 for Cry1Ac activated toxin (Table 1). Because the resistance ratio was similar for Cry1Ac protoxin and activated toxin, these results suggest that reduced activation of Cry1Ac protoxin was not a major mechanism of resistance in LF120 relative to LF. We can compare the results here with previous results from LF and the strains derived progressively from LF that were selected in the laboratory with Cry1Ac protoxin to achieve progressively higher resistance: LF5 (least resistant), LF10, LF20, LF30, LF60, and LF120 (most resistant) [29, 40–42]. Consistent with the results here, Chen et al. reported that LF120 was highly resistant to Cry1Ac activated toxin (resistance ratio = 1700 in that study versus 1200 in this study, Table 1) [42]. Also consistent with our results, Xiao et al. found that the LF60 strain, the immediate parent strain from which LF120 was derived, had a similar resistance ratio for Cry1Ac protoxin (1400) and Cry1Ac activated toxin (1100) [41]. For both LF60 and LF120 (Table 1), the LC_50_ value did not differ significantly between Cry1Ac protoxin and activated toxin [41]. The resistance ratio for Cry1Ac protoxin divided by the resistance ratio for Cry1Ac activated toxin was 1.3 for LF120 (Table 1) and 1.3 for LF60 [41]. These results imply that decreased activation of Cry1Ac protoxin had a similar, minor role in resistance of both of these strains.

By contrast with LF120 and LF60, for the LF5 strain derived directly from LF, the LC_50_ value was significantly higher for Cry1Ac protoxin than activated toxin, and the resistance ratio was 2.8 times higher for Cry1Ac protoxin (110) than activated toxin (39) [29]. The resistance in LF5 is genetically linked with a mutation that decreases transcription of the trypsin gene *HaTryR* by 99% and reducing transcription of this gene in susceptible LF larvae with RNA interference doubled the survival on diet treated with Cry1Ac protoxin [29]. The results imply that production of the trypsin protease encoded by *HaTryR* is important for activation of Cry1Ac protoxin. Transcription of the four other trypsin genes examined did not differ between LF and LF5 [29]. Because LF5 was a precursor strain of LF120, the mutation causing reduced transcription of *HaTryR* in LF5 might also occur in LF120 and contribute to its resistance to Cry1Ac protoxin. Although it remains to be determined if this mutation occurs in LF120, our results do show that the activity of trypsin-like proteases were greater in LF than LF120 (Fig 5), consistent with the idea that decreased activity of trypsin-like proteases contributed to resistance in LF120.

In contrast to the association between resistance and reduced trypsin-like activity, the activity of chymotrypsin-like proteases did not differ significantly between LF and LF120 (Fig 5). Consistent with these results, reduced chemotrypsin activity was not associated with the resistance of LF10, LF20, and LF30 relative to LF5 [40].

As expected, the trypsin inhibitor TLCK decreased activation of Cry1Ac protoxin by trypsin and the chymotrypsin inhibitor TPCK decreased activation of Cry1Ac protoxin by chymotrypsin (Figs 1 and 2). However, neither TLCK nor TPCK significantly increased the LC_50_ of Cry1Ac protoxin against LF or LF120 (Table 2), which implies that blocking only trypsin-like proteases or only chymotrypsin-like proteases did not have a major effect on toxicity of Cry1Ac protoxin. In addition, activation of Cry1Ac protoxin by midgut extract from LF larvae was reduced by TLCK, but not by TPCK (Figs 3 and 4), which indicates the reduction in activation achieved was greater by blocking trypsin-like proteases with TLCK than by blocking chymotrypsin-like proteases with TPCK. Nonetheless, the decrease in activation caused by TLCK was greater and lasted longer for trypsin (Fig 1) than for the midgut extract (Fig 3), which implies that proteases other than trypsin contribute to activation of Cry1Ac protoxin by the midgut extract.

Overall, the evidence reported here and from related studies [29, 40–42], indicates that in LF120 and the other resistant strains of *H*. *armigera* derived by laboratory selection from the susceptible LF strain, reduced activation of Cry1Ac protoxin associated with lower activity of trypsin-like proteases is a minor mechanism of resistance. In particular, reduced activation of Cry1Ac protoxin cannot explain the >1000-fold resistance to Cry1Ac activated toxin in the LF60 and LF120 strains (Table 1) [41, 42]. Accordingly, other mechanisms probably account for most of the resistance in these two strains. For example, mis-splicing of the gene encoding ABCC2 is linked with resistance to Cry1Ac in LF60 [41]. The results reported here and previously from the resistant strains derived from LF mirror the patterns seen with other strains of *H*. *armigera* and other insects, where high levels of resistance are most commonly caused by mutations that disrupt binding of Cry proteins to midgut target sites [32–33].

## Materials and Methods

### Insects

The susceptible LF strain was started with 200 larvae collected from Langfang County, Hebei Province of China in 1998 and reared in the laboratory without exposure to Bt toxins or insecticides. The resistant LF120 strain was derived from the LF strain via selection of a series of progressively more resistant strains: LF5, LF10, LF20, LF30, LF60, and LF120 [29, 40–42, 43]. Each resistant strain was selected by diet incorporation of MVPII (Dow AgroSciences), a commercial formulation containing a hybrid protoxin similar to CryAc protoxin [44]. The concentration for selection for each strain corresponds with the number from 5 to 120 following LF in the strain name. LF120 was selected at 120 μg Cry1Ac protoxin per ml of diet [42]. All larvae were reared on artificial diet. The insects were maintained at 27 ± 1°C, 60 ± 10% relative humidity (RH), and a photoperiod of 14L:10D h [45].

### Cry1Ac protoxin and activated toxin

Cry1Ac protoxin crystals were obtained from the HD-73 strain of *B*. *thuringiensis* (kindly supplied by Biotechnology Research Group, Institute of Plant Protection, Chinese Academy of Agricultural Sciences). The Cry1Ac protoxin was prepared as described by Wang et al. [46]. To active Cry1Ac, at 37°C for 6 h in 10 ml, pH10.0, 50 mM Na_2_CO_3_ buffer with 0.4 mg of trypsin from bovine pancreas (SIGMA) (25:1 protein concentration). Activated Cry1Ac was then precipitated by adding 4 M acetic acid dropwise to the activation reaction tube to adjust pH to 4.5, incubating at 4°C for 15 min, and centrifuge at 10,000 x g for 15 min. The upper supernatant was transferred into a clean tube and used as the stock solution for bioassays. the concentrations of Cry1Ac protoxin or activated Cry1Ac toxin in the stock solution were estimated by electrophoresis of 10 μl 250 μg/ml BSA solution as well as 10 μl of Cry1Ac protoxin or activated Cry1Ac toxin stock solution on a SDS–PAGE gel and quantification of the intensity of the corresponding bands with Image J software [47].

### Diet bioassays

We used surface overlay diet bioassays [48] with seven concentrations of Cry1Ac protoxin or activated toxin against each strain: 0 to 96 ng/cm^2^ diet against LF and 0 to 56,700 ng/cm^2^ diet for LF120. We diluted Cry1Ac with 50 mM pH 10.0 Na_2_CO_3_ buffer, and the buffer alone was used as a control. Sixty μL of each dilution was applied to the surface of the artificial diet that had been dispensed in 24-well plates and allowed to cool. When the surface of the diet was dry, one neonate was placed in each well. Each 24-well plate constituted one replicate, and three replicates were used per treatment, yielding a total of 504 larvae tested for each bioassay. Mortality was recorded after 7 d.

### Effects of the trypsin inhibitor TLCK and chymotrypsin inhibitor TPCK in bioassays

To test effects of the protease inhibitors TLCK and TPCK (Sigma Chemical Compnay), we dissolved 10 mg of each inhibitor in 1 ml DMSO (dimethylsulfoxide) and applied 1 microliter of each dilution to the dorsum of two-day-old larvae [49]. In these experiments DMSO without inhibitor was applied to the dorsum of two-day-old larvae. After treatment with TLCK, TPCK, or DMSO without inhibitor, larvae were immediately used in bioassays as described above.

### Cry1Ac protoxin activation by trypsin and chymotrypsin

We activated Cry1Ac protoxin with bovine trypsin (Sigma Chemical Company) alone, with trypsin and the trypsin inhibitor TLCK, with chymotrypsin (alpha-chymotrypsin, AMRESCO) alone, and with chymotrypsin and the chymotrypsin inhibitor TPCK. We tested each inhibitor at ratios of 10 mg protease: 1 mg inhibitor or 1 mg protease: 1 mg inhibitor. We added each mixture of protease and inhibitor to Cry1Ac protoxin at the rate of 1 mg protease: 100 mg Cry1Ac protoxin to digest for 30 min and 2 h. We separated the digested Cry1Ac protein by 8% SDS-PAGE, stained with Coomassie brilliant blue R250, and analyzed band intensity using the Image J software [47].

### Cry1Ac protoxin activation by midgut extracts

We activated Cry1Ac protoxin with midgut extracts from LF and LF120 larvae alone and with protease inhibitors. We prepared LF midgut extract as described below and Cry1Ac activation and analysis as described above. Three biological replicates were used in this experiment. 100 μg of Cry1Ac protoxin (1 mg/mL) in pH 10.0 Na_2_CO_3_ buffer was incubated with 1 μL NaCl (negative control) or midgut extract from LF and LF120 (1 μg total protein per reaction) at 37°C for 30, 60, 120 and 160 min, respectively. Then the reactions were stopped by adding 25 μL 5 × SDS-PAGE loading dye and boiling for 10 min, and separated on a 8% SDS-PAGE gel at 120 V for 1.5 h.

### Midgut extracts

We prepared midgut extracts from LF and LF120 as described by Wang and Qin [50]. In brief, 10 midguts of early fifth instars were dissected and homogenized in 1.5 ml of homogenization buffer (0.15 M NaCl) on ice as one biological replicate. The homogenate was centrifuged at 4°C and 10,000 g for 15 min to remove debris [40, 51]. The supernatant was collected, and the protein concentration was determined with the Bradford method using a Protein Assay kit (Pierce, Rockford, IL) and BSA as the protein standard [52]. Three technical replicates were used to determine the protein concentration. All midgut extracts were diluted to 1 mg/ml (1mg proteins per 1 ml 0.15 M NaCl) before being used in the activity and activation assays.

### Protease activity

We tested the protease activity of midgut extracts from LF and LF120. We used BApNA (α-benzoyl-DL-arginine-p-nitroanilide) and SAAPFpNA (N-succinyl-alanine-alanine- proline-phenylalanine-p-nitroanilide) (Sigma Chemical Company) as substrates to study the activitiy of trypsin-like proteases and chymotrypsin-like proteases, respectively. We prepared midgut extracts as described above. We mixed 10 μl of midgut extracts (1 mg protein per ml 0.15 M NaCl) with 45 μl of glycine and sodium hydroxide buffer (pH 10.0) [40, 50]. The activity of trypsin-like protease was determined by adding 100 μl BApNA (1 mg/ml). For chymotrypsin-like protease, 5 μL of midgut solution was mixed with 90 μl glycine and sodium hydroxide buffer (pH 10.0) [40, 50]. The activity of chymotrypsin-like protease was determined by adding 45 μl of SAAPFpNA (1 mg/ml). The absorbance at 405 nm was monitored for 15 min, with measurements taken every 10 seconds [40, 50]. The enzyme activity was expressed as the change in absorbance per minute per mg of protein for each midgut extract. We conducted three biological replicates per strain.

### Induction of proteases in LF larvae by Cry1Ac

We used 10 early fifth instars of LF for each treatment with three biological replicates per treatment. After larvae were starved for 12 h, we put them on diet containing a range of 0 to 13.3 μg Cry1Ac protoxin per g diet. After larvae were on the diet for 12 h, we dissected their midguts, prepared midgut extracts, and used the methods described above to measure the protease activity of the midgut extracts.

### Statistical analysis

We calculated the LC_50_, its 95% fiducial limits, and slope of the concentration-mortality using probit analysis [53]. We calculated the resistance ratio (RR) by dividing the LC_50_ for LF120 by the LC_50_ of LF. We calculated the inhibition ratio (IR) by dividing the LC_50_ from the treatment with inhibitor (e.g., Cry1Ac protoxin + TLCK) by the LC_50_ from the treatment without inhibitor (e.g., Cry1Ac protoxin alone). The molecular weight was 130 kDa for Cry1Ac protoxin and ca. 65 kDa for activated Cry1Ac protoxin, as quantified with the Image J software [47]. The percentage of Cry1Ac protoxin activated was calculated by dividing the optical density of the activated toxin band of each treatment by the optical density of the protoxin band of the negative control on the same gel. We used t-tests [54] to evaluate differences between strains in protease activity and activation of Cry1Ac protoxin. Cry1Ac protoxin activation data were arcsine transformed before analysis. We used linear regression [55] to evaluate the association between the concentration of Cry1Ac in diet and activity of trypsin-like proteases and chymotrypsin-like proteases in fifth instars of the susceptible LF strain.

## Supporting Information

S1 Fig Cry1Ac increases activity of proteases in fifth instars from the susceptible LF strain of *H*. *armigera*. Mean and standard error (SE) are shown from three replicates. (A) Trypsin-like proteases. Linear regression: R^2^ = 0.99, df = 3, P = 0.00048. (B) Chymotrypsin-like proteases. Linear regression: R^2^ = 0.99, df = 3, P = 0.00053.
(TIF)

S1 Table Data for Fig 1. Activation of Cry1Ac protoxin by trypsin with and without the trypsin inhibitor TLCK.
(DOCX)

S2 Table Data for Fig 2. Activation of Cry1Ac protoxin by chymotrypsin with and without the chymotrypsin inhibitor TPCK.
(DOCX)

S3 Table Data for Fig 3. Activation of Cry1Ac protoxin by midgut extract with and without the trypsin inhibitor TLCK.
(DOCX)

S4 Table Data for Fig 4. Activation of Cry1Ac protoxin with and without the chymotrypsin inhibitor TPCK.
(DOCX)

S5 Table Data for Fig 5. Protease activity in LF and LF120 strain of *H*. *armigera*.
(DOCX)

S6 Table Data for Fig 6. Time course of the activation of Cry1Ac protoxin by midgut extracts from LF and LF120 strain of *H*. *armigera*.
(DOCX)
