# Supplementary figures and images for: Activation of Bt Protoxin Cry1Ac in Resistant and Susceptible Cotton Bollworm
Source: PLoS One. 2016 Jun 3;11(6):e0156560. doi: 10.1371/journal.pone.0156560 (PMC4892611; doi:10.1371/journal.pone.0156560)

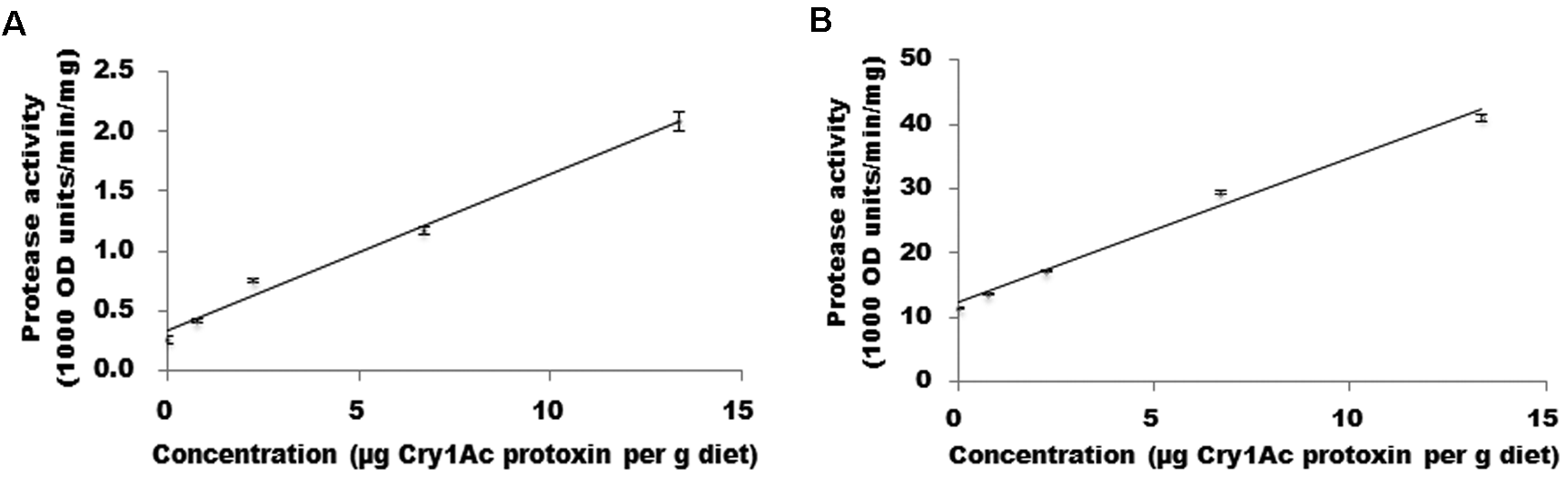

Supplement: S1 Fig — Mean and standard error (SE) are shown from three replicates. (A) Trypsin-like proteases. Linear regression: R2 = 0.99, df = 3, P = 0.00048. (B) Chymotrypsin-like proteases. Linear regression: R2 = 0.99, df = 3, P = 0.00053. (TIF) [file pone.0156560.s001.tif]
