# Supplementary material for: Activation of Bt Protoxin Cry1Ac in Resistant and Susceptible Cotton Bollworm
Source: PLoS One. 2016 Jun 3;11(6):e0156560. doi: 10.1371/journal.pone.0156560 (PMC4892611; doi:10.1371/journal.pone.0156560)
Supplement: S1 Table — Activation of Cry1Ac protoxin by trypsin with and without the trypsin inhibitor TLCK. (DOCX) [file pone.0156560.s002.docx]

**S1 Table. Data for Fig 1. Activation of Cry1Ac protoxin by trypsin with and without the trypsin inhibitor TLCK.**

| 30 min | Percentage activation of Cry1Ac protoxin (%) | | |
| --- | --- | --- | --- |
|  | Repeat 1 | Repeat 2 | Repeat 3 |
| Cry1Ac protoxin and trypsin (lane 3) | 100 | 100 | 100 |
| Cry1Ac protoxin and 10:1 trypsin + TLCK (lane 4) | 12.58 | 12.60 | 11.00 |
| Cry1Ac protoxin and 1:1 trypsin + TLCK(lane 5) | 8.77 | 7.51 | 7.49 |
| 2 h |  |  |  |
| Cry1Ac protoxin and trypsin (lane 6) | 100 | 100 | 100 |
| Cry1Ac protoxin and 10:1 trypsin + TLCK(lane 7) | 12.01 | 13.12 | 13.99 |
| Cry1Ac protoxin and 1:1 trypsin + TLCK (line 8) | 11.20 | 11.80 | 11.20 |
