# Supplementary material for: Activation of Bt Protoxin Cry1Ac in Resistant and Susceptible Cotton Bollworm
Source: PLoS One. 2016 Jun 3;11(6):e0156560. doi: 10.1371/journal.pone.0156560 (PMC4892611; doi:10.1371/journal.pone.0156560)
Supplement: S2 Table — Activation of Cry1Ac protoxin by chymotrypsin with and without the chymotrypsin inhibitor TPCK. (DOCX) [file pone.0156560.s003.docx]

**S2 Table. Data for Fig 2. Activation of Cry1Ac protoxin by chymotrypsin with and without the chymotrypsin inhibitor TPCK.**

| 30 min | Percentage activation of Cry1Ac protoxin (%) | | |
| --- | --- | --- | --- |
|  | Repeat 1 | Repeat 2 | Repeat 3 |
| Cry1Ac protoxin and chymotrypsin (lane 3) | 100 | 100 | 100 |
| Cry1Ac protoxin and 10:1 chymotrypsin + TPCK (lane 4) | 45.00 | 44.08 | 62.00 |
| Cry1Ac protoxin and 1:1 chymotrypsin + TPCK (lane 5) | 33.32 | 21.55 | 33.55 |
| 2 h |  |  |  |
| Cry1Ac protoxin and chymotrypsin (lane 6) | 100 | 100 | 100 |
| Cry1Ac protoxin and 10:1 chymotrypsin + TPCK (lane 7) | 87.00 | 93.33 | 83.44 |
| Cry1Ac protoxin and 1:1 chymotrypsin + TPCK (line 8) | 81.15 | 73.48 | 66.62 |
