# Supplementary material for: Activation of Bt Protoxin Cry1Ac in Resistant and Susceptible Cotton Bollworm
Source: PLoS One. 2016 Jun 3;11(6):e0156560. doi: 10.1371/journal.pone.0156560 (PMC4892611; doi:10.1371/journal.pone.0156560)
Supplement: S3 Table — Activation of Cry1Ac protoxin by midgut extract with and without the trypsin inhibitor TLCK. (DOCX) [file pone.0156560.s004.docx]

**S3 Table. Data for Fig 3. Activation of Cry1Ac protoxin by midgut extract with and without the trypsin inhibitor TLCK.**

| 30 min | Percentage activation of Cry1Ac protoxin (%) | | |
| --- | --- | --- | --- |
|  | Repeat 1 | Repeat 2 | Repeat 3 |
| Cry1Ac protoxin and midgut extract (lane 3) | 85.03 | 88.22 | 88.78 |
| Cry1Ac protoxin and 10:1 midgut extract + TLCK (lane 4) | 56.70 | 68.90 | 78.00 |
| Cry1Ac protoxin and 1:1 midgut extract + TLCK (lane 5) | 52.09 | 67.00 | 53.00 |
| 2 h |  |  |  |
| Cry1Ac protoxin and midgut extract (lane 6) | 100 | 100 | 100 |
| Cry1Ac protoxin and 10:1 midgut extract + TLCK (lane 7) | 97.97 | 87.70 | 92.30 |
| Cry1Ac protoxin and 1:1 midgut extract + TLCK (line 8) | 93.02 | 78.99 | 88.01 |
