# Supplementary material for: Activation of Bt Protoxin Cry1Ac in Resistant and Susceptible Cotton Bollworm
Source: PLoS One. 2016 Jun 3;11(6):e0156560. doi: 10.1371/journal.pone.0156560 (PMC4892611; doi:10.1371/journal.pone.0156560)
Supplement: S4 Table — Activation of Cry1Ac protoxin with and without the chymotrypsin inhibitor TPCK. (DOCX) [file pone.0156560.s005.docx]

**S4 Table. Data for Fig 4. Activation of Cry1Ac protoxin with and without the chymotrypsin inhibitor TPCK.**

| 30 min | Percentage activation of Cry1Ac protoxin (%) | | |
| --- | --- | --- | --- |
|  | Repeat 1 | Repeat 2 | Repeat 3 |
| Cry1Ac protoxin and midgut extract (lane 3) | 88.45 | 94.05 | 79.55 |
| Cry1Ac protoxin and 10:1 midgut extract + TPCK (lane 4) | 86.35 | 78.80 | 81.75 |
| Cry1Ac protoxin and 1:1 midgut extract + TPCK (lane 5) | 65.90 | 89.00 | 71.01 |
| 2 h |  |  |  |
| Cry1Ac protoxin and midgut extract (lane 6) | 100.00 | 100.00 | 100.00 |
| Cry1Ac protoxin and 10:1 midgut extract + TPCK (lane 7) | 86.82 | 95.90 | 83.18 |
| Cry1Ac protoxin and 1:1 midgut extract + TPCK (line 8) | 78.99 | 74.25 | 90.01 |
