# Supplementary material for: Activation of Bt Protoxin Cry1Ac in Resistant and Susceptible Cotton Bollworm
Source: PLoS One. 2016 Jun 3;11(6):e0156560. doi: 10.1371/journal.pone.0156560 (PMC4892611; doi:10.1371/journal.pone.0156560)
Supplement: S5 Table — Protease activity in LF and LF120 strain of H. armigera. (DOCX) [file pone.0156560.s006.docx]

**S5 Table. Data for Fig 5. Protease activity in LF and LF120 strain of *H. armigera*.**

| Trypsin-like ODmin-1mg-1) |  |  |  |
| --- | --- | --- | --- |
|  | Repeat 1 | Repeat 2 | Repeat 3 |
| LF | 393 | 498 | 408 |
| LF120 | 62 | -34 | 12 |
| chymotrypsin-like ODmin-1mg-1) |  |  |  |
| LF | 5061 | 4424 | 4436 |
| LF120 | 3452 | 3563 | 3483 |
