# Supplementary material for: Activation of Bt Protoxin Cry1Ac in Resistant and Susceptible Cotton Bollworm
Source: PLoS One. 2016 Jun 3;11(6):e0156560. doi: 10.1371/journal.pone.0156560 (PMC4892611; doi:10.1371/journal.pone.0156560)
Supplement: S6 Table — Time course of the activation of Cry1Ac protoxin by midgut extracts from LF and LF120 strain of H. armigera. (DOCX) [file pone.0156560.s007.docx]

**S6 Table. Data for Fig 6. Time course of the activation of Cry1Ac protoxin by midgut extracts from LF and LF120 strain of *H. armigera.***

| Treatments | Percentage activation of Cry1Ac protoxin (%) | | |
| --- | --- | --- | --- |
|  | Repeat 1 | Repeat 2 | Repeat 3 |
| LF,30min | 86.37 | 68.45 | 84.73 |
| LF,60min | 96.87 | 76.60 | 94.82 |
| LF,120min | 99.18 | 66.45 | 82.26 |
| LF,180min | 99.95 | 68.95 | 82.86 |
| LF120,30min | 53.95 | 42.05 | 52.05 |
| LF120,60min | 74.80 | 57.48 | 71.15 |
| LF120,120min | 87.33 | 69.47 | 86.00 |
| LF120,180min | 99.67 | 66.94 | 85.35 |
